# Exosomes: The role in mammalian reproductive regulation and pregnancy-related diseases

**DOI:** 10.3389/fphys.2023.1056905

**Published:** 2023-03-10

**Authors:** Xing-Ru Guo, Yun Ma, Zi-Ming Ma, Tian-Shu Dai, Shi-Hao Wei, Yuan-Kui Chu, Xin-Gang Dan

**Affiliations:** ^1^ School of Agriculture, Ningxia University, Yinchuan, Ningxia, China; ^2^ Department of Laboratory Medicine, General Hospital of Ningxia Medical University, Ningxia Medical University, Yinchuan, China

**Keywords:** exosome, pregnancy-related diseases, embryo implantation, follicular development, endocrine regulation

## Abstract

Exosomes are a kind of extracellular vesicles that are produced and secreted by different mammalian cells. They serve as cargo proteins and can transfer different kinds of biomolecules, including proteins, lipids, and nucleic acids, which consequently act on target cells to exert different biological effects. Recent years have witnessed a significant increase in the number of studies on exosomes due to the potential effects of exosomes in the diagnosis and treatment of cancers, neurodegenerative diseases, and immune disorders. Previous studies have demonstrated that exosomal contents, especially miRNAs, are implicated in numerous physiological processes such as reproduction, and are crucial regulators of mammalian reproduction and pregnancy-related diseases. Here, we describe the origin, composition, and intercellular communication of exosomes, and discuss their functions in follicular development, early embryonic development, embryonic implantation, male reproduction and development of pregnancy-related diseases in humans and animals. We believe this study will provide a foundation for revealing the mechanism of exosomes in regulating mammalian reproduction, and providing new approaches and ideas for the diagnosis and treatment of pregnancy-related diseases.

## 1 Introduction

Extracellular vesicles (EVs) that are released by different kinds of cells, mediate cell communication between tissues and organs. EVs can be classified into microvesicles, apoptotic vesicles, and exosomes according to their characteristics and functions. Exosomes, a special subtype of extracellular vesicles with a diameter of approximately 30 nm–150 nm (Jeppesen et al., 2019), function as carriers of information molecules for intercellular communication. In addition, they participate in different physiological and pathological responses (Farooqi et al., 2018).

Exosomes were first identified in sheep reticulocytes, and little was known about their functions (Johnstone et al., 1987). Substantial research in recent years has revealed the functions and significance of exosomes in physiological and pathological processes. For instance, Valadi et al. (2007) found that nucleic acid substances, such as mRNA and miRNA in the exosomes of mouse mast cells, can regulate the biological functions of other cells. Similarly, other studies have demonstrated that exosomes carrying miRNAs, IncRNAs, and circRNAs, can be transported to specific tissues and organs *via* body fluids and play an important role in regulating physiological functions (Zheng et al., 2020). In addition, exosomes participate in several pathological processes, such as tumorigenesis, the development of neurodegenerative diseases, and immune disorders.

## 2 Mammalian exosomes: Their origin and composition

Almost all cells, including epithelial cells, macrophages, mast cells, neuronal cells, and mesenchymal cells, can secrete exosomes. Furthermore, immune cells can secrete exosomes, which play an important role in the immune response during tumorigenesis by influencing the proliferation and activity of receptor cells (Homer et al., 2017; Chung et al., 2020). Moreover, exosomes have been identified from body fluids, such as cerebrospinal fluid, saliva, serum, breast milk, and urine (Nguyen et al., 2016).

**FIGURE 1:**
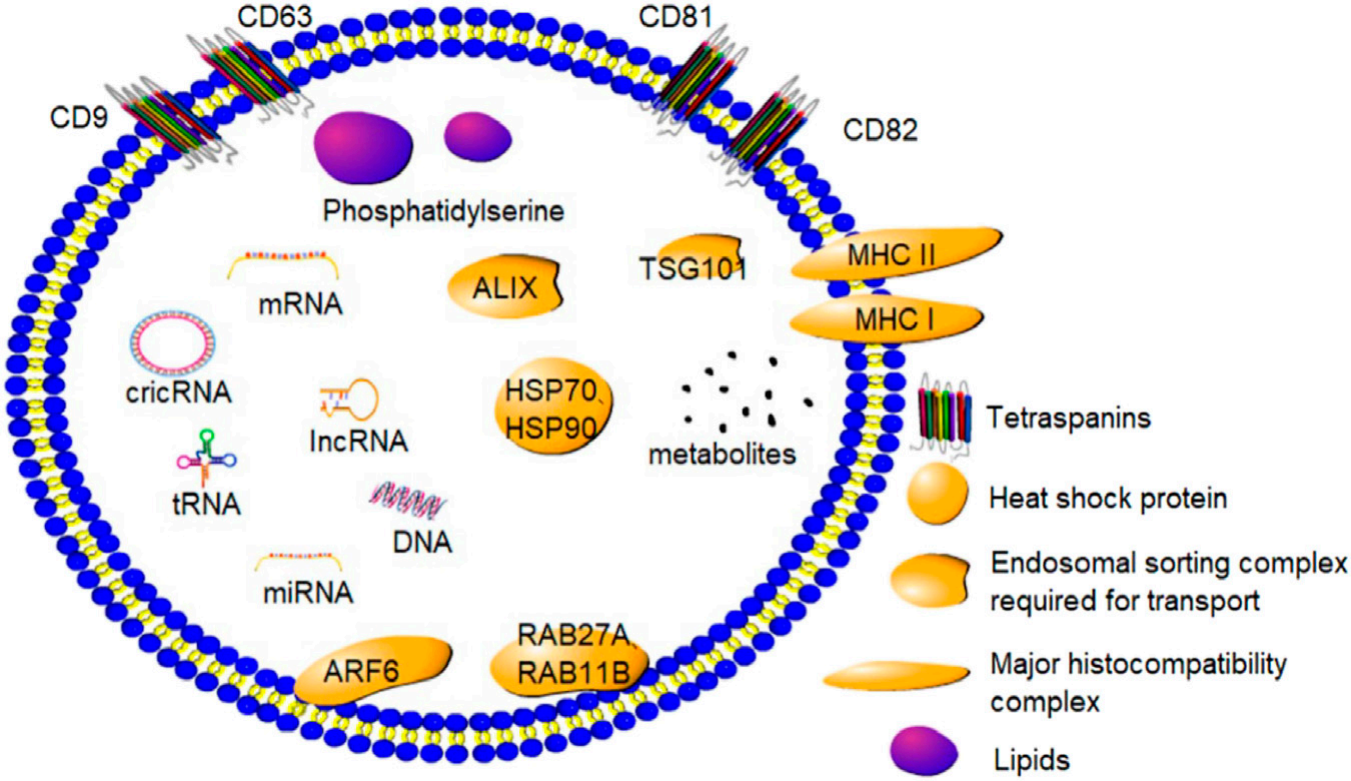
Composition and marker proteins of exosomes: Exosomes are small phospholipid bilayer vesicles that contain a variety of proteins, lipids, and nucleic acids. CD9, CD63, CD81, and CD82 are marker proteins of exosomes.

Exosomes have a complicated biological composition, with several of them containing distinct components and different species possessing different exosomes. Exosomes primarily contain proteins, lipids, and nucleic acids (Figure 1). The majority of exosomal proteins are essential proteins although certain unique proteins produced during exosome formation are also present. Exosomes contain tetra-transmembrane proteins, namely, CD9, CD63, CD81, and CD82, which are now considered to be the marker proteins of exosomes (Pluchino and Smith, 2019). Furthermore, exosomes are enriched with endonuclear somatic sorting complex proteins (ALIX and TSG101), heat shock proteins (HSP70 and HSP90), and certain special proteins responsible for the formation of extracellular vesicles and their release, such as RAB27A, RAB11B, and ARF6 (Kurian and Modi, 2019). Moreover, exosomes are rich in glycoproteins and transmembrane proteins, including EGFR, MHC I, MHC II, and other transmembrane proteins (Zaborowski et al., 2015).

Exosomes are a rich source of different kinds of lipids. The exosome membrane is largely composed of sphingolipids, gangliosides, and unsaturated lipids, with a low content of phosphatidylcholines and diglycerides. In addition, ganglioside-rich GM3 and ceramide derivatives such as phosphatidylserine have been found in the exosomes (Llorente et al., 2013). The composition of exosomes changes in response to changes in the extracellular environment (Kalluri and LeBleu, 2020).

Since the discovery of mRNAs and miRNAs in the exosomes, different kinds of exosomal biomolecules have been identified. Several studies have demonstrated that exosomes contain different kinds of small RNAs, including ribosomal 18 S and 28 S subunit rRNAs and certain tRNAs. In a study, rRNA was found to be a major biomolecule of exosomes secreted by human mammary cell lines (Jenjaroenpun et al., 2013). In addition, lncRNA and circRNA have been located in the exosomes (Abels and Breakefield, 2016). These exosomal biomolecules can bind to target cells to govern several key physiological functions in animals.

## 3 Exosome formation and intercellular communication

**FIGURE 2:**
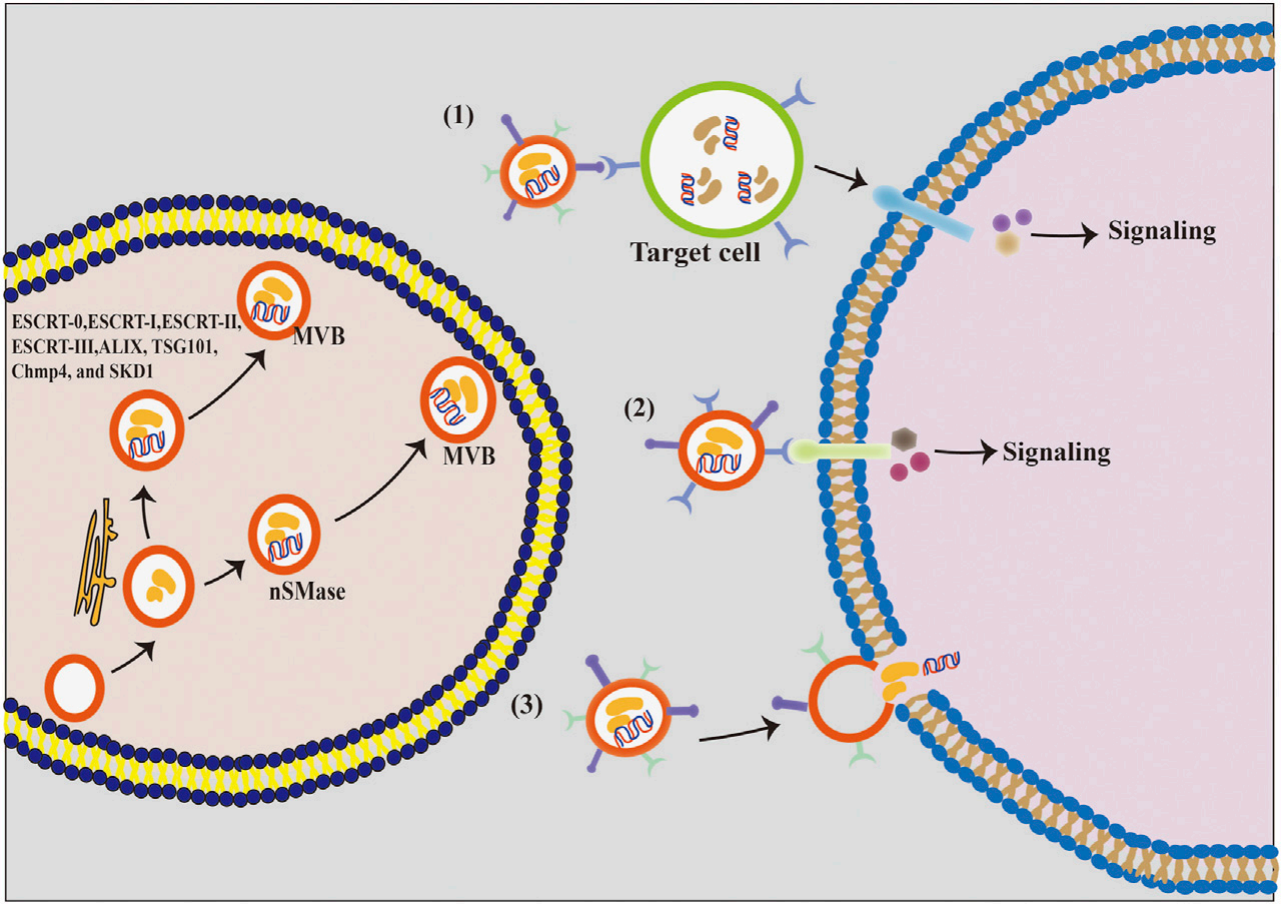
The formation process and the intercellular communication mode of exosomes. (1) Exosomal membrane protein first binds to the cellular receptor, and then acts on its target cells and activates the intracellular signaling cascades. (2) Exosomal membrane proteins bind directly to the target cell’s receptors and activate their intracellular signaling pathways. (3) Exosomal membrane directly fuses with the phospholipid bilayer of the target cell, and exosomal components, including proteins, lipids, nucleic acids, and other substances, are released into the cytoplasm of the target cell in a non-selective manner.

Exosomes are synthesized by the double invagination of the protoplasmic membrane and the inward budding of the luminal membrane of intracellular multivesicular bodies (MVBs) containing intraluminal vesicles (ILVs, Figure 2). The initial invagination of the plasma membrane leads to the formation of early sorting endosomes, including cellular proteins, lipids, metabolites, small molecules, and ions (Urbanelli et al., 2013). Subsequently, the second invagination allows early sorted endosomes to mature into late sorted endosomes, eventually producing MVBs containing ILVs. The formation of exosomes is largely controlled by two mechanisms, namely, the ESCRT-dependent mechanism and the ESCRT-independent mechanism (van Niel et al., 2018). ESCRT-dependent formation of MVBs is a mono-ubiquitination modification of membrane proteins and occurs by the addition of ubiquitin molecules to lysine residues recognized by ESCRT proteins and ESCRT-related proteins, including ALIX, TSG101, Chmp4, and SKD1. These proteins bind to the ubiquitinated vesicles to form MVBs. In contrast, the ESCRT-independent formation of MVBs is facilitated by neutral sphingomyelinase (nSMase), which promotes membrane invagination of ILVs and the formation of MVBs. Finally, MVBs merge with lysosomes and autophagosomes to degrade biomolecules or merge with the plasma membrane to release the exosomes (Abels and Breakefield, 2016; Jeppesen et al., 2019).

Exosomes play a crucial ‘postman’ function in the extracellular environment by efficiently delivering the information molecules to certain target cells, thereby regulating the activities of target cells (Carp et al., 2004; Cui et al., 2012). It is currently believed that exosomes communicate with cells in three ways (Figure 2). First, exosome vesicles adhere to the surface of the recipient cells by interacting with the lipid or ligand–receptor, subsequently activating numerous signaling pathways of the effector cell. Second, exosomes can directly trigger the target cells and activate the related signaling pathways. Third, exosomes release biomolecules into the cytoplasm of target cells after fusing with their plasma membranes, thereby activating these cells (Wei et al., 2021). Therefore, exosomes can regulate several physiological functions in animals through different communication ways.

## 4 Exosomes regulate mammalian reproduction

Exosomal biomolecules, including non-coding RNAs, hormones, and proteins, have been implicated in regulating human and animal reproduction. Exosomes can transport RNAs, proteins, enzymes, and lipids, thereby affecting multiple physiological and pathological processes in several diseases, including cancer, neurodegenerative diseases, infections, and autoimmune diseases. Exosomal bioinformatic molecules of different origins can regulate their physiological functions by acting on the corresponding target tissues. Exosomes in the follicular fluid carrying miRNA, mRNA, and proteins can communicate bidirectionally with granulosa cells and oocytes to regulate granulosa cell proliferation, steroid production, and oocyte maturation (Collado-Fernandez et al., 2012; Yuan C et al., 2021). Besides, exosomes are released from the uterus, oviduct epithelium, endometrium, and preimplantation embryos and are known to regulate follicle development, embryo development, and implantation (Machtinger et al., 2016; Qamar et al., 2020). Endometrium can regulate the process of embryo attachment by secreting exosomes into the uterine cavity fluid, the embryo itself can also secrete exosomes during embryo implantation. Exosomes secreted by the embryo have been reported to alter the morphology of the endometrium and its gene expression profile, thereby regulating embryo positioning and invasion for embryo implantation (Kusama et al., 2018). Furthermore, exosomal miRNAs and proteins in the placenta are known to regulate the inflammatory response and trophoblast invasion (Gurunathan et al., 2022). Exosomes derived from embryonic and uterine mucosa can mediate immune tolerance through mutual communication with the mother, facilitating embryo implantation and subsequent pregnancy (Wu Y et al., 2022). Recent studies have demonstrated that intrauterine EV-derived bta-miR-26b and miR-98 downregulate the maternal immune system, which contributes to the implantation of the conceptus (Nakamura et al., 2019; Nakamura et al., 2021). Exosomal biomolecules in the reproductive system mediate bidirectional communication between cells and tissues and regulate different physiological events. Of these, exosomes regulating follicular and embryonic development and embryo implantation have attracted the highest attention of researchers. In addition, extensive evidence has revealed the role of exosomes in the male genital tract. Recently, exosomes derived from testicular cells not only regulate normal spermatogenesis and steroidogenesis in the testis but can also be used as a diagnostic marker for male infertility (Ma et al., 2022).

### 4.1 Exosomes regulate follicles and early embryonic development

Granulosa cells are essential for follicular development. Follicular exosomal biomolecules are known to regulate granulosa cell steroid production, proliferation, and apoptosis. Human umbilical cord MSC-derived EVs promote estrogen production of granulosa cells (Cai et al., 2022). Another study involving patients with polycystic ovarian syndrome (PCOS) reported that the deletion of exosomal circLDLR in receptor cells enhanced the expression of miR-1294 and inhibited that of CYP19A1, which consequently inhibited estradiol secretion and ultimately resulted in aberrant follicular growth (Huang et al., 2020). In contrast, Yao et al. (2010) reported that exosomal miR-224 stimulated estradiol secretion and promoted follicular growth by increasing the expression of CYP19A1 mRNAs. This suggests that different exosomal biomolecules exert varying regulatory effects on the same target tissue. In addition, exosomal miRNAs can affect follicular development by regulating the proliferation and apoptosis of ovarian granulosa cells. Bovine follicular fluid-derived EVs promote the proliferation of granulosa cells (Andrade et al., 2017). However, miR-424 in follicular fluid EVs from patients with PCOS inhibits granule proliferation by targeting CDCA4 to inhibit the Rb/E2F1 signaling (Yuan D et al., 2021). In addition, granulosa apoptosis can cause follicular developmental arrest and atresia. Xu et al. (2017) demonstrated that exosomal miR-145 in follicular fluid protected mouse granulosa cells from oxidative stress-induced apoptosis and suppressed aberrant follicular development by targeting the KLF4 receptor. Another study reported that miR-92a in follicular fluid exosomes targets SMAD7 to inhibit the apoptosis of porcine granulosa cells and maintain follicular development. MiR-23a and miR-27a from follicular fluid exosomes can target SMAD5 to promote the apoptosis of human ovarian granulosa cells *via* the FasL–Fas signaling pathway, ultimately causing follicular atresia (Nie et al., 2015). This suggests that different exosomal molecules in follicular fluid carrying different communication signals can influence follicular development by regulating granulosa cell functions.

In addition, exosomes have been implicated in the development of oocytes. It has been previously demonstrated that bovine follicular exosomes can enhance oocyte development by promoting the proliferation of cumulus cells (Hung et al., 2015). Yang et al. (2020) claimed that miR-146a-5p or miR-21–5p carried by exosomes isolated from human umbilical cord mesenchymal stem cells promoted oocyte development and improved the number and quality of mouse oocytes *via* the PI3K/mTOR signaling pathway. Similarly, exosomal miR-17- and miR-92a-derived follicular fluid increased the oocyte diameter and H4K12 acetylation levels (Inoue et al., 2020). On the contrary, miR-145 suppressed oocyte development by reducing the levels of neurotrophic factors (Xu et al., 2017). Subsequently, increased exosomal miRNAs regulating the oocyte development were identified using bioinformatic approaches. Hu et al. (2020a) identified miR-125b, let7d-5p, miR-200b, miR-26a, and miR-92a in porcine follicular fluid exosomes by next-generation sequencing, and reported that they could be involved in the regulation of the maturation of the nucleus and cytoplasm of pig oocytes *via* the TGF signaling pathway. Thus, during the development of the oocyte, exosomes in the follicle carry several information molecules into different cells to coordinate the communications among cells, thereby contributing to the growth and maturation of the oocyte.

Furthermore, follicular fluid exosomes can promote zygote division and blastocyst development. Exosomes have been found to promote the proliferation of cumulus cells and the development of the embryo as well as reduce the side effects of heat shock on blastocyst cleavage and development (Rodrigues et al., 2019). Furthermore, exosomal miRNA-21 is known to modulate the growth and development of the zygote and suppress embryonic death (Lv et al., 2018). Supplementation of EVs with small follicles (3–6 mm) during the development of embryos in vitro culture altered the levels of embryonic transcripts associated with epigenetic modifications and embryonic development, and altered the overall DNA methylation and hydroxymethylation levels, thereby increasing blastocyst rates (Da et al., 2017). This suggests that follicular fluid-derived EVs contribute to early embryonic development. Although exosomal biomolecules are known to regulate follicular and embryonic development, the exact underlying molecular mechanism is still poorly understood.

### 4.2 Exosomes regulate the process of mammalian embryo implantation

**FIGURE 3:**
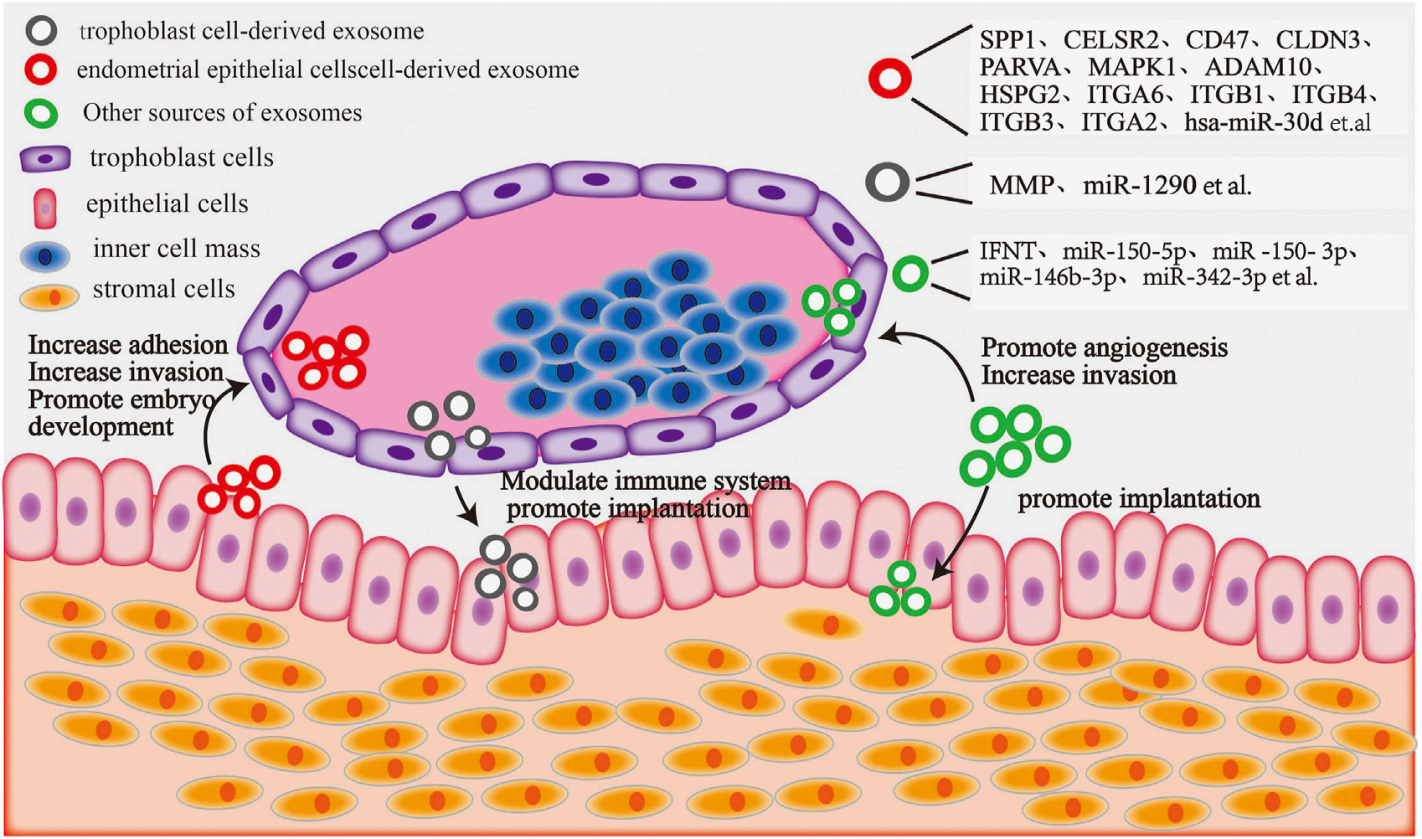
The blastocyst and endometrium interact with exosome components: endometrial epithelial cell-derived exosomal proteins and miRNAs can target trophoblast cells, increasing their adhesion and invasion and promoting embryo development. Embryonic trophoblast cell-derived exosomal proteins and miRNAs can regulate the maternal immune system and increase embryo attachment by targeting endometrial epithelial cells. Moreover, exosomal components derived from other sources (including plasma, serum, and follicular fluid) can target trophoblast cells to facilitate invasion and angiogenesis, whereas others can target endometrial epithelial cells to promote embryonic implantation.

The exchange of information between the endometrium and the embryo is indispensable for successful embryo implantation (Salomon et al., 2016). For instance, exosomal proteins are necessary for mutual communication between the embryo and the endometrium during the early stages of pregnancy, facilitating embryo implantation. Exosomes derived from endometrial epithelial cells are taken up by embryonic trophoblast cells during embryo implantation and secrete specific proteins, thereby enhancing the adhesion of trophoblast cells to the endometrium and promoting embryo implantation. Exosomal miRNAs isolated from the supernatant of cultured porcine endothelial cell lines have been reported to regulate the function of trophoblast cells (Bidarimath and Tayade, 2017). Moreover, in pigs, recent studies have demonstrated that miR-92b-3p, an exosome derived from porcine endometrial epithelial cells, is engulfed by porcine trophectoderm cells and regulates the proliferation, migration, and adhesion of trophoblast cells by targeting TSC1 and DKK3 (Hua et al., 2022). Another study reported that Hsa-miR-30d, an exosomal message molecule released from human endometrial cells, is engulfed by mouse embryos to regulate embryo implantation (Vilella et al., 2015). In addition, other miRNAs in the exosomes can increase the adhesion of endometrial epithelial cells and the invasiveness of blastocyst trophectoderm during embryo implantation (Gurung et al., 2020). This suggests that the regulatory function of homologous exosomal informative molecules is conserved between species. Not only are exosomal miRNAs important informative molecules that regulate embryo implantation but exosomal proteins also play an important role in embryo implantation. Osteopontin, also known as secreted phosphoprotein 1 (SPP1), is an extracellular matrix protein that binds to integrin receptors on porcine and sheep trophectoderm cells (Johnson et al., 2014). SPP1 has been reported to mediate trophoblast adhesion in the endometrium by activating the mTOR, PI3K, MAPK3/MAPK1, and MAPK14 signaling pathways. Furthermore, exosomal proteins from trophoblast cells are involved in maternal–fetal recognition during bovine pregnancy. In addition, the variations in exosomal proteins regulate endometrial receptivity (Su et al., 2021). Certain proteins can regulate embryo implantation in the form of both secreted proteins and exosomal informative molecules. Interferon-tau (IFNT) is known to be the main trophoblast factor acts on the endometrium, inhibiting luteolysis and maintaining progesterone secretion. Imakawa et al. (2017) reported that endogenous retroviruses in maternal exosomes promote the growth of trophoblast ectodermal cells and release IFNT *via* binding to Toll-like receptors, thus inducing the expression of adhesion molecules and interferon-stimulated genes during early sheep gestation. Subsequently; Kusama et al. (2018) showed that IFNT is not only a secretory protein stimulated by exosomes but also an exosomal component in the endometrium of pregnant ruminants; Salomon et al. (2017) reported that trophoblast cell-derived exosomes promote extrachorionic trophoblast infiltration and embryonic implantation by activating the expression of matrix metalloproteinases (MMPs) and protein kinase MAPK signaling pathways. Endometrium-derived exosomes MMP-14 could degrade and remodel the extracellular matrix in the embryo–endometrium and promote embryo implantation (Latifi et al., 2018). Proteins and miRNAs contained in different sources of exosomes play an important role in the interaction between blastocyst and endometrium during embryo implantation (Figure 3). These findings suggest that biomolecules carried by exosomes promote the adhesion and implantation of embryos; however, the exact regulatory mechanisms warrant further investigations.

In addition, exosome-derived biomolecules can be employed as novel indicators to evaluate endometrial receptivity and embryo implantation in mammals. Bioinformatic analysis revealed that 348 endometrial exosomal miRNAs were identified as possible biomarkers of endometrial receptivity and fertility in mammals (Altmae et al., 2017). Similarly, Zhou et al. (2020) stated that exosome-specific miRNAs in peripheral blood are potential tools for determining embryonic implantation. Furthermore, Zeng et al. (2021) reported that miR-150–5p, miR-150–3p, miR-146b-3p, and miR-342–3p in plasma exosomes are involved in embryo implantation, whereas miR-146b-3p and miR-150–5p serve as diagnosis targets of embryo implantation. Moreover, exosomal miR-17 and miR-20a, belonging to the miR-17/92 family can exchange trophoblast invasion and embryo implantation by targeting TGF-receptor II, Smad2, and Smad4 (Mogilyansky and Rigoutsos, 2013). The trophoblast-derived exosomal miR-1290 can suppress the expression of LHX6 and promote the epithelial–mesenchymal transition, thereby enhancing endometrial receptivity (Shi et al., 2021). In all, the miR-17/92 cluster and miR-1290 are potential novel biomarkers for evaluating endometrial receptivity and embryo implantation. However, further studies are required to assess endometrial tolerance and embryo implantation using exosomal informative molecules.

### 4.3 Exosomes regulate male reproduction

In addition to regulating female reproduction, the role of exosomes in male reproduction has recently received increasing attention. Some studies have demonstrated that exosomes derived from male genital tract including Sertoli cell, epididymis and prostate are involved in germ cell development, sperm maturation and fertilization outcomes (Baskaran et al., 2020). Recent study showed that exosomal miR-486–5p derived from Sertoli cell acts as a communication molecule between Sertoli cells and spermatogonial stem cell (SSCs), modulating differentiation of SSCs through binding *PTEN* in mice (Li et al., 2021). Further study also demonstrated that exosomes released Sertoli cells may cross the blood-testis barrier and promote the survival of Leydig cells (Ma et al., 2022). It is well known that seminal plasma is essential for the maturation of spermatozoa. Meanwhile, exosomal biomolecules containing protein and miRNA in seminal plasma regulate a range of biological processes involving maturation of spermatozoa in the epididymis. A study has indicated that seminal exosomes could promote sperm capacitation through inducing acrosome reaction (Murdica et al., 2019a). Yang et al. (2017) identified 1,474 proteins in male seminal plasma exosomes, and found that these proteins were mainly involved in biologic processes, including metabolism, energy pathways, protein metabolism, cell growth and maintenance. Another study also found a total of 2,138 proteins isolated from seminal plasma of normozoospermic (NSP) and severe asthenozoospermic (SA) men by comprehensive proteomic analysis, of which 37 miRNAs were increased in the NSP group and 52 miRNAs were increased in the SA group, supporting the evidence that the network of seminal exosomes proteins may be connected to the molecular mechanisms involved in sperm maturation and motility (Murdica et al., 2019b). In animals, boar seminal plasma exosomes were essential in maintaining sperm motility, sperm membrane integrity, antioxidant capacities and inhibition of premature capacitation (Du J et al., 2016). In addition to various exosomal proteins, non-coding RNAs, including microRNA, Y RNA and tRNA derived from seminal plasma are able to modulate sperm function. Sperm cytoplasmic droplets (CDs) are remnants of cytoplasm, and their migration is a morphological characteristic of epididymal maturation. Sun et al.(2021) identified total of 348 known and 206 new miRNAs in boar seminal plasma exosomes isolated from semen containing spermatozoa with CDs, of which 13 miRNA were significantly upregulated, whereas three miRNAs were significantly downregulated, suggesting that seminal plasma exosomes might play a key role in the regulation of sperm CDs. Currently, semen exosomes are also able to play an important role in male mammalian reproduction as biomarker candidates for the identification of patients with azoospermia, hypospermia and teratozoospermia or other male infertility (Candenas and Chinease, 2020). Furthermore, extracellular adenosine triphosphate (exATP) produced by boar seminal plasma exosomes may control sperm motility by regulating mitochondrial metabolism (Guo et al., 2019). Further study indicated that fresh boar sperm incubated with exATP significantly increased sperm motility and reduced apoptotic rate (Guo et al., 2019). Most recently, using seminal plasma exosomes isolated from the seminal plasma of a bull of proven fertility could also improve the fertilizing capacity of male gametes of low-fertility bulls (Lange-Consiglio et al., 2022). These suggest that exosomes have some potential improving male reproduction. However, isolation and purification of exosome derived from male genital tract still presents a substantial hurdle (Goss et al., 2023).

## 5 Exosomes role in the diagnosis and treatment of pregnancy-related diseases

### 5.1 Exosomes in diagnosing pregnancy-related diseases

Although exosomes are required for mammalian pregnancy, variations in exosome contents can induce several pregnancy-related diseases. It has been recently reported that the abnormal expression of exosomal proteins and non-coding RNAs is related to pregnancy-related diseases. For instance, patients with preeclampsia have considerably higher levels of miR-136, miR-494, and miR-495 in their peripheral blood exosomes compared to those in 20 weeks of pregnancy peripheral blood, indicating that these miRNAs could serve as biomarkers for early diagnosis of preeclampsia (Motawi et al., 2018). Two clinical studies demonstrated that miR-21 and miR-200 families (miR-200a, miR-200b, and miR-200c) in female serum exosomes can serve as human ovarian cancer biomarkers (Mahmoud et al., 2018). Zhang et al. (2020a) reported that the expression of exosomal miR-22–3p and miR-320a significantly increased in patients with endometriosis, and speculated that these miRNAs can be used as potential biomarkers for the diagnosis of endometriosis. Another study reported that exosomal proteins extracted from the peritoneal fluid of endometriosis patients, such as PRDX1, H2A type 2-C, ANXA2, and ITIH4 and tubulin α-chain, which are absent in the peritoneal fluid of healthy women, could be used as biomarkers to identify endometriosis (Nazri et al., 2020). Furthermore, differential expression analysis of serum exosomes revealed relatively higher miR-27a-5p levels in PCOS patients than in normal women (Che et al., 2020). Therefore, miR-27a-5p levels of circulating exosomes could serve as promising biomarkers for PCOS. Another frequent pregnancy problem is pregnancy loss. A comparison of the differences in blood exosomes between women with normal pregnancy and those with recurrent pregnancy loss revealed that the expression of exosome marker CD9 significantly increased in women with recurrent pregnancy loss at the sixth week of pregnancy (Rajaratnam et al., 2021). Similar to studies on humans, certain exosomal miRNAs can be employed as markers to identify early pregnancy loss in cows. For instance, the expression of miR-25, miR-16b, and miR-3596 carried serum exosomes was higher in early aborting cows, indicating that exosomal miRNAs could trigger luteum regression and cause pregnancy loss (Pohler et al., 2017). Similarly, De Bem et al. (2017) reported reduced levels of 27 miRNAs in peripheral blood from cattle with early embryonic loss. Therefore, these exosomal miRNAs could be used as diagnostic markers for early pregnancy loss in humans and animals. In addition, other exosomal components could be valuable biological targets for the diagnosis of pregnancy-associated abnormalities in humans and animals (Table 1).

**TABLE 1:** Differential exosomes as biomarkers of pregnancy-related disease.

Diseases	Species	Biomarkers	Type	References
Preeclampsia	human	miR-136, miR-494, miR-495	miRNA	Motawi et al. (2018)
Endometriosis	human	miR-22–3p, miR-320	miRNA	Zhang et al. (2020b)
	human	miR-26b-5p, miR-215–5p, miR-6795–3p	miRNA	Wu H. M et al. (2022)
	human	miRNA-15a-5p	miRNA	Wu et al. (2021)
	human	has-miR-6745	miRNA	Lu and Gao (2021)
	human	PRDX1, H2A type 2-C, ANXA2, ITIH4, tubulin α-chain	Protein	Nazri et al. (2020)
	human	aHIF	lncRNA	Qiu et al. (2019)
	human	tRF-Leu-AAG-001	siRNA	Li et al. (2022)
	human	Circ_0026129	circRNA	Wu et al. (2021)
pregnancy loss	human	CD9	Protein	Rajaratnam et al. (2021)
	cow	miR-25, miR -16b, miR -3,596	miRNA	Pohler et al. (2017)
	cow	bta-miR-499	miRNA	De Bem et al. (2017)
Polycystic Ovary Syndrome	human	miR-25–3p, miR-143–3p, miR-193b-3p, miR-199a-5p, miR-6087, miR-10a-5p, miR-23b-3p, miR-98–5p, miR-199a-3p, miR-199b-3p, miR-629–5p, miR-4532, miR-483–5p, miR-483–3p, miR-3911, miR-4745–3p, miR-141–3p, miR-200a-3p, miR-200c-3p, miR-382–5p	miRNA	Hu et al. (2020b)

### 5.2 Exosomes in the treatment of pregnancy-related diseases

An increasing number of studies have demonstrated the potential of exosomes in treating pregnancy-related diseases. The exosome-delivered miR-323–3p (exosomes of adipose mesenchymal stem cells from PCOS patients) promoted the proliferation and inhibited apoptosis in CCs by targeting PDCD4, indicating its potential role in the treatment of PCOS (Vojtech et al., 2014; Zhao et al., 2019). Moreover, circLDLR in the follicular fluid exosomes reduced the formation of aberrant follicles in PCOS by targeting miR-1294, suggesting circLDLR is a novel target for future PCOS treatment (Huang et al., 2020). Another study demonstrated that exosomal miR-424–5p exerted a therapeutic impact on PCOS patients *via* inhibiting granulosa cell proliferation and inducing PCOS cell senescence (Yuan C et al., 2021). Subsequently, different exosomal miRNAs have been demonstrated to be effective in preventing and treating PCOS. Adipose mesenchymal stem cell-derived exosomal miRNA-21–5p increased the number of corpus luteum and reduced the number of abnormal cystic follicles by targeting Btg2 and activating the IRS1 and AKT signaling pathways in PCOS rats (Cao et al., 2022). In addition, Zhou et al. (2022) demonstrated that the overexpression of miR-18b-5p derived from follicular fluid reduced the apoptosis of granulosa cells and attenuated the pathological effects in ovaries by targeting PTEN and activating the PI3K/Akt/mTOR signaling pathway in PCOS rats.

Endometriosis is a common disease in human and animal pregnancy. Exosomal components are involved in regulating the development of endometriosis. For example, Liu et al. (2021) found that the expression of lncRNA CHL1-AS1 promoted the proliferation, migration, and invasion of endometrial stromal cells and inhibited cell apoptosis, thereby causing endometriosis in the mouse. Another study reported the involvement of several exosomal miRNAs in the development of endometriosis by reducing the expression of HOXA10 and LIF in women (Zhou and Dmitridis, 2020). Moreover, endometriosis usually causes tissue fibrosis. Exosomal miR-214–3p inhibited the development of endometriosis fibrosis by directly targeting connective tissue growth factors (Zhang et al., 2021; Li et al., 2022). Furthermore, exosomal miR-30c from endometrial epithelial cells inhibited the invasion and migration of ectodermal endometrial epithelial cells by counter-regulating the expression of BCL9, implying miR-30c to be a potential therapeutic target in endometriosis (Zhang et al., 2022). These findings suggest that exosomal biomolecules regulate endometriosis, and these could be used to design a novel treatment method for endometriosis.

Pregnancy loss can occur due to a disturbance in between the anti-inflammatory and pro-inflammatory states at the maternal–fetal interface. The placenta-derived exosome bta-miR-499 inhibited LPS-induced inflammation in cultured bovine endometrial epithelial cells, and lower levels of mmu-miR-499 have been shown to increase the risk of bovine pregnancy loss *in vivo* (Zhao et al., 2018). Therefore, miR-499 can serve as a potential therapeutic target for pregnancy loss caused by uterine inflammation. Another study reported that peritoneal injection of exosomal granulocyte–colony-stimulating factor derived from trophoblast cells significantly reduced the rate of miscarriage in mice, suggesting it could be a novel therapeutic regimen for pregnancy loss (Gao et al., 2022). Furthermore, injecting mesenchymal stem cells-derived exosomes into the uterine horn of mice downregulated the levels of IFN-γ and TNF-α, and upregulated the levels of IL-10 and IL-4, suggesting its potential role in reducing embryonic resorption and maintaining normal pregnancy (Xiang et al., 2020). Therefore, specific tissue-derived exosomes can be used for the management of pregnancy loss. However, we need to identify more exosomal molecules and further investigate their potential mechanisms of action.

In addition, exosomes derived from different tissues can be used to treat other pregnancy-related diseases. A study on patients with endometrial cancer reported that exosome efficiently transported miR-148b to endometrial cancer cells *via* fibroblasts, where the miRNA inhibited endometrial cancer metastasis by directly binding to its target gene DNMT1 (Li Q et al., 2019). Wang et al. (2021) reported that down-regulating exosomal miR-218 expression in endometrial epithelial cells of cows with endometritis impaired embryonic development and reduced trophoblast migration by inhibiting the WNT signaling pathway. Consequently, an injection of exosomal miR-218 reduced endometritis-induced infertility in cows. Other exosomes have been reported to be involved in the development and treatment of pregnancy-related diseases in both humans and animals as shown in Table 2. Although exosomes regulate the occurrence of mammalian diseases and have shown potential value in treating pregnancy-related diseases, further studies are required to completely understand the underlying mechanisms.

**TABLE 2:** Exosomes associated with reproductive diseases in different mammals.

Disease	Species	Main components of exosome contents	Type	Main function	Signaling pathways	References
Preeclampsia	human	miRNA-136, miRNA-494, miRNA-495	miRNA	Inhibition of angiogenesis, inhibition of vascular endothelial growth factor	—	Motawi et al. (2018)
	human	miR-153–3p, miR-653–5p, miR-325, miR-222–3p	miRNA	Inhibition of cell proliferation and invasion of human umbilical vein endothelial cells	PI3K/AK and ERK signaling pathways	Li B. L et al. (2019)
	human	miR-18b-3p	miRNA	Reduce systolic blood pressure and proteinuria, reduce serum inflammatory factors	—	Gong et al. (2021)
	human	lncRNA TUG1	lncRNA	Reduce trophoblast proliferation and inhibit angiogenesis	—	Li et al. (2020a)
	human	lncRNA H19	lncRNA	Inhibition of trophoblast apoptosis	—	Huang Y et al. (2021)
	human	syncytin-1, syncytin-2	Protein	Inhibition of trophoblast apoptosis	—	Vargas et al. (2014)
Ovarian Cancer	human	miR-223	miRNA	Promotion of drug resistance in ovarian cancer cells	PTEN-PI3K/AKT signaling pathway	Zhu et al. (2019)
Endometrial Cancer	human	miR-148b	miRNA	Inhibition of epithelial-mesenchymal transition in endometrial cancer cells	—	Li Q et al. (2019)
	human	miR-320a	miRNA	Inhibition of endometrial cancer cell proliferation	HIF1 α/VEGFA signaling pathway	Zhang et al. (2020)
	human	miR-21	miRNA	Promotion of endometrial cancer invasion	—	Xiao et al. (2020)
	human	lectin galactoside-binding soluble 3 binding protein (LGALS3BP)	Protein	Promotes cell proliferation and migration of endometrial cancer cells	PI3K/AKT/VEGFA signaling pathway	Song et al. (2021)
Endometritis	dairy cattle	AC_000161_317, bta-miR-708, bta-miR-91b bta-let-7d, bta-miR-1, bta-miR-122, bta-miR-146a, bta-miR-3600, bta-miR-451	miRNA	Impeding the development of blastocyst	—	Wang et al. (2019)
	dairy cattle	miR-218	miRNA	Impedes embryonic development and reduces placental trophoblast cell migration	—	Wang et al. (2021)
Endometrial Fibrosis	rat	lncRNA-MIAT	lncRNA	Alleviation of fibrosis after endometrial injury	—	Shao et al. (2021)
Gestational Diabetes	human	miR-520h	miRNA	Promotion of trophoblast apoptosis	mTOR signaling pathway	Wen and Bai (2021)
	rat	miR-26b	miRNA	Acceleration of gestational diabetes progression	PI3K/Akt signaling pathway	Li et al. (2020b)
	human	miR-16–5p	miRNA	Resulting in abnormal Wnt/β-linked protein signaling	PI3K/Akt, Wnt, and mTOR signaling pathways	Herrera-Van et al. (2020)
	human	circ_0074673	circRNA	Inhibition of proliferation, migration and angiogenesis of human umbilical vein endothelial cells	—	Huang Q et al. (2021)

## 6 Conclusions and perspectives

Although exosomes can be used as potential markers for the early diagnosis of diseases, early pregnancy, and miscarriage detection, their extraction is highly time-consuming and expensive. Therefore, a simple, rapid, and inexpensive method is required to efficiently extract exosomes in the future. In addition, exosomal components are complex, and current research is focused on miRNAs and proteins. However, research on other exosomal components, such as mRNAs and lipids, is very limited and warrants further investigation. Exosomes can cross biological barriers and transport numerous proteins and gene-based drugs into target cells. Certain aspects that limit their use as drug carriers are as follows: First, exosomes cannot be stored indefinitely, and the present protective measures could alter exosome morphology and impair their carrier function. Thus, future studies should concentrate on developing more effective preservation approaches to preserve exosome’s biological activity. Second, the targeting ability of natural exosomes is poor and they are easily cleared *in vivo*. This problem can be overcome using genetic engineering techniques or chemical linking of targeting peptides. Third, the majority of studies on exosomes are *in vitro* due to the lack of suitable animal models. Therefore, establishing suitable animal models of specific diseases will help conduct further studies on exosomes. Fourthly, immature techniques for the isolation and purification of exosome have severely limited its clinical use. Yet, it is encouraging that microfluidic technology has the potential to simplify and improve exosome isolation and detection.

Altogether, this review highlights the biological functions of exosomes in different physiological events associated with reproduction and pregnancy-related diseases. The composition, origin, and communication of exosomes have been described in detail. Numerous studies have demonstrated that exosomes carrying functional molecules can regulate different physiological events such as follicle development, oocyte maturation, granulosa cell function, and embryo implantation. In addition, exosomal informative molecules are involved in regulating the development of pregnancy-related diseases, some of which can be used as new biological targets for the diagnosis of pregnancy-related diseases. Exosomes delivering specific target drugs have demonstrated potential clinical applications in the treatment of pregnancy-related diseases. Although certain shortcomings in exosome research cannot be effectively solved at present, it is believed that the research value of exosomes in regulating reproduction and the diagnosis and treatment of mammalian pregnancy-related diseases will increase tremendously with the development of research technologies.
